# Helping small babies survive: an evaluation of facility-based Kangaroo Mother Care implementation progress in Uganda

**DOI:** 10.11604/pamj.2014.19.37.3928

**Published:** 2014-09-16

**Authors:** Patrick Aliganyira, Kate Kerber, Karen Davy, Nathalie Gamache, Namaala Hanifah Sengendo, Anne-Marie Bergh

**Affiliations:** 1 Saving Newborn Lives programme, Save the Children, Kampala, Uganda and Washington DC, USA; 2 University of the Western Cape, Bellville, South Africa; 3 MRC Unit for Maternal and Infant Health Care Strategies, University of Pretoria, South Africa

**Keywords:** Delivery of health care, implementation, infant premature, Kangaroo Mother Care, neonatal, newborn, preterm, program evaluation, Uganda

## Abstract

**Introduction:**

Prematurity is the leading cause of newborn death in Uganda, accounting for 38% of the nation's 39,000 annual newborn deaths. Kangaroo mother care is a high-impact; cost-effective intervention that has been prioritized in policy in Uganda but implementation has been limited.

**Methods:**

A standardised, cross-sectional, mixed-method evaluation design was used, employing semi-structured key-informant interviews and observations in 11 health care facilities implementing kangaroo mother care in Uganda.

**Results:**

The facilities visited scored between 8.28 and 21.72 out of the possible 30 points with a median score of 14.71. Two of the 3 highest scoring hospitals were private, not-for-profit hospitals whereas the second highest scoring hospital was a central teaching hospital. Facilities with KMC services are not equally distributed throughout the country. Only 4 regions (Central 1, Central 2, East-Central and Southwest) plus the City of Kampala were identified as having facilities providing KMC services.

**Conclusion:**

KMC services are not instituted with consistent levels of quality and are often dependent on private partner support. With increasing attention globally and in country, Uganda is in a unique position to accelerate access to and quality of health services for small babies across the country.

## Introduction

Prematurity is the leading cause of newborn death in Uganda, accounting for 38% of the nation's 39,000 annual newborn deaths [1]. After pneumonia and malaria it is the third leading cause of deaths amongst children under age five [1]. Over 200,000 or 14% of Ugandan babies are born prematurely (before 37 weeks gestation) [2]. Those who survive may face a lifetime of disability with limited access to supportive services. These figures illustrate the urgency of addressing this burden and echo what is seen as an emerging priority in global public health [3]. Approaches to improve quality of care of preterm infants in health facilities were ranked second out of 82 questions in global research priority setting for preterm babies [4].

One of the highest impact interventions for newborn survival and health is kangaroo mother care (KMC) [5]. It is a low-tech and cost-effective intervention in which mothers serve as human “incubators” for their newborns. KMC comprises a set of care practices for low birth weight newborns - including continuous skin-to-skin contact, establishing breastfeeding, and close follow up after discharge from a health facility [6]. KMC has been shown to reduce neonatal mortality by over 50% amongst babies weighing less than 2000g at birth [7]. It has also been found to be highly effective in reducing severe morbidity, particularly from infection [8]. Other effects when compared with incubator care include the reduction of hypothermia, severe illness, lower respiratory tract disease, and length of hospital stay. Babies cared for with KMC show improved weight gain, length, and head circumference, breastfeeding, and mother-infant bonding [8, 9]. Despite convincing evidence, KMC uptake is low and only a very small proportion of newborns who could benefit from KMC receive it [10].

Compared to some other countries in the region, KMC was highlighted in Uganda at a late stage. KMC was first introduced in Mulago Hospital in 2001, but there was very little further spread of the practice beyond this national level teaching hospital. KMC remained “under the radar” while a more comprehensive approach to policy change encompassing a broader package of facility and community interventions for newborn survival was adopted nationally [11].

One of the events in Uganda that brought KMC into the public domain was a provocative editorial cartoon and newspaper article from August 7, 2007 entitled “Government tells mothers to use charcoal stoves as makeshift incubators” [12]. According to this article, the Director-General of Health Services recommended the use of the *sigiri*, a charcoal stove, to keep premature babies warm in poor, rural areas. This was followed by a period of advocacy for more appropriate methods of thermal care. On August 29, 2007 articles on KMC were published in both national daily newspapers in which the KMC method and its advantages were explained [13, 14].

According to a newborn situation analysis commission by the Ministry of Health and overseen by the country's national newborn steering committee, a number of major challenges for newborn health were identified and included the limited availability of special services such as KMC for the care of preterm babies at health centre level, “inadequate knowledge of newborn care among health providers, a lack of institutional support for evidence-based low-cost interventions, such as KMC, and a critical lack of trained staff” [15]. Hence preterm babies born at home or at lower levels of care were almost always referred to hospitals and if referral was not possible, lanterns and coal stoves were used to provide extra heat in the rooms. Even in the hospitals, locally made incubators were used but they were prone to breakdown and suboptimal functioning due to irregular power supply [15]. As a result, the national newborn steering committee recommended immediate action at health facility level to increase the speed of roll-out of KMC in facilities starting at the health-centre level IV and above, with strong links to community follow up [15].

An evaluation of KMC services in Uganda was undertaken in order to gauge the progress towards scaling up KMC following these recommendations. The evaluation aimed at systematically measuring the scope and institutionalisation of KMC services and to describe barriers and facilitators to sustainable KMC services.

## Methods

A cross-sectional, mixed-method evaluation design was used to analyse the country's progress with KMC implementation against a previously developed stages-of-change model [16] which has been used elsewhere [17, 18]. Approval to conduct the evaluation was obtained from the Ministry of Health, Uganda and ethics approval obtained by the Institutional Review Board of the Johns Hopkins School of Public Health. Both qualitative and quantitative data collection methods were employed, including semi-structured key-informant interviews, observations and a review of quantitative secondary data.

Sampling took place in two phases. First, stakeholders involved in newborn care activities across the country were convened to solicit their views and perceptions of the provision of KMC and their expectations of the evaluation. Second, a convenience sample of 11 out of the 17 health care facilities across the country that were reported to provide KMC services at the time of the study were targeted for a personal visit. KMC focal persons and other staff and members of the management team working in these facilities provided the necessary “grass roots” information needed for measuring progress in KMC implementation. The 11 facilities visited included one national/referral teaching hospital, one regional hospital, 4 district hospitals, 2 health centres IV, and 3 private-not-for-profit hospitals.

Facilities were assessed by two progress monitors by means of standardized, key-informant interviews and an observation inventory covering the following aspects of service and types of practices: the health care facility (including its baby-friendly status); neonatal and KMC facilities; skin-to-skin practices; history of KMC implementation; involvement of internal role players; physical and financial resources; KMC space (continuous and intermittent KMC); feeding and weight monitoring; referral, discharge and follow up; record keeping and documentation; KMC education; staffing issues (orientation and training; rotations); strengths and challenges [16]. Each facility received a score out of a total of 30. The scoring is divided according to six stages of institutionalisation, with each stage having a weighted score: create awareness (2 points); commit to implement (2 points); prepare to implement (6 points); implement (7 points); integrate into routine practice (7 points); sustain practice (6 points) [16].

## Results

Assessed facilities differed in terms of the level of institutionalisation of KMC services; the resources and support available for KMC; on-site KMC practices; and procedures around discharge and follow-up care.

### KMC implementation progress and level of institutionalization

**Figure 1:**
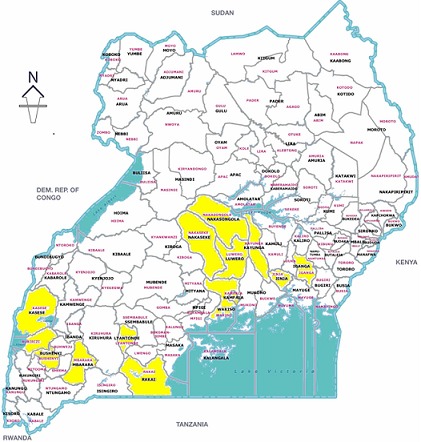
Map of Uganda showing districts reporting at least one KMC facility

Facilities with KMC services are not equally distributed throughout the country. Only four regions (Central 1, Central 2, East-Central and Southwest) plus the City of Kampala were identified as having facilities providing KMC services (Figure 1).

**Figure 2:**
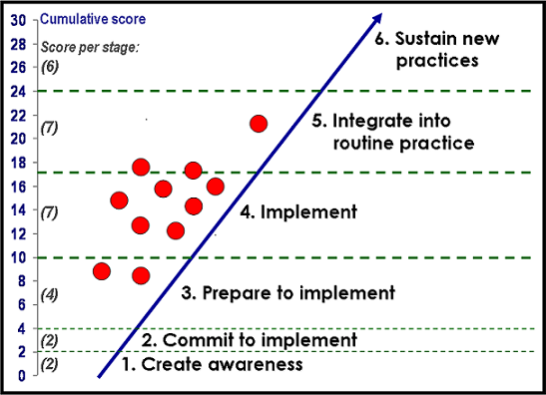
Status of KMC institutionalisation

The facilities visited scored between 8.28 and 21.72 out of the possible 30 points (Table 1). The mean score of the total of facilities was 14.45 and the median score 14.71. According to the scoring metric applied, two of the assessed facilities were in the process of taking ownership of the concept of KMC (scores of 8.28 and 9.25), 3 were on the road to KMC practice (scores of 12.16, 12.53 and 14.03), 5 facilities showed evidence of KMC practice (scores of 14.71, 15.78, 16.15, 17.07 and 17.33) (Figure 2). Only one facility demonstrated evidence of institutionalised practice (score of 21.72). The hospital with the lowest score was a facility with a history of starting KMC in 2006, then it stopped and KMC was re-introduced in 2010. Two of the 3 highest scoring hospitals were private, not-for-profit hospitals with a Christian mission background, whereas the second highest scoring hospital was a central teaching hospital.

**Table 1:** Facility scores and interpretation of the scores

Facility	Score	Interpretation	Number & type of facility
1	8.28	In the process of taking ownership of the concept of KMC (Stage 3)	1 regional hospital 1 district hospital*
2	9.25		
3	12.16	On the road to KMC practice (Stage 4)	1 private hospital** 2 district hospitals*
4	12.53		
5	14.03		
6	14.71	Evidence of KMC practice (Stage 4)	2 health centres IV 1 district hospital 1 private hospital** 1 central hospital
7	15.78		
8	16.15		
9	17.07		
10	17.33		
11	21.72	Evidence of institutionalised practice (Stage 5)	1 private hospital**

* Health centres IV recently upgraded to district hospitals

** Private-not-for-profit hospitals

### Resources and support for KMC implementation

In terms of physical space provided for KMC, 6 facilities provided a special space for KMC mothers and babies. Four facilities had separate rooms with 1-6 beds, a corner separated by curtains in one and a broad corridor in another. The rest of the facilities had designated beds in the postnatal ward.

According to the impressions of the assessors, there was a lot of involvement of senior management in the implementation of KMC in the case of 4 facilities, with some involvement in 5 facilities and no involvement or a neutral position in 2 facilities. Hospital directors, in collaboration with medical superintendents and head nurses were reported to be supportive by allowing a variety of staff members to be trained in essential newborn care and KMC, by giving material support (e.g. space alterations) and by showing personal interest. As one informant remarked, “*He (director) was there for us - support and was in all meetings*.” Nursing managers were also reported to have been actively involved in the initiation of KMC and in the sensitisation of hospital staff.

Though KMC does not require much in terms of materials, this was raised as a concern. For the most part mothers provided their own materials to tie the baby in the KMC position. In one facility, linen was provided but not laundry service. Private, external support played a significant role in the start-up and continuation of KMC services. Each of the assessed health facilities reported the provision of resources in the form of training, formative supervision after training and/or equipment and materials as part of the introduction of KMC. These resources were reported to have been provided by partners including Save the Children, UNICEF, Uganda Private Midwives Association, ISIS Foundation, and Rotary International (Table 2). An informant from one hospital described the absence of internal support, “*So far I've only seen Save (the Children)*.”

**Table 2:** Overview of support with equipment and materials

Partner	No. of facilities	Types of equipment and materials*
Save the Children	8	Stationery (books and register) Equipment (digital baby scales, chairs) Educational materials (posters, flipcharts) Building materials and curtains Supportive resources (KMC wraps, booties, cap, blanket for mother)
Rotary International (different groups)	3	Linen General neonatal care equipment (ambubags, incubators)
Uganda Professional Midwives Association (UPMA)	1	Resuscitation equipment Bed sheets and blankets Drug dispenser and some drugs
ISIS Foundation**	1	Equipment (feeding cups and spoons, digital baby scale, comfortable KMC chairs) Supportive resources (KMC wraps) Building of neonatal unit Food for mothers General neonatal care equipment (cots, incubators)
UNICEF	1	Newborn protocols
Volunteer doctor	1	Material for wraps Plastic chairs
Uganda Newborn Survival Study (UNEST)	1	General labour ward equipment (labour ward beds and sheets, BP machines, bulb syringes, cannulas) Materials for infection control (cleaning equipment, bleach, gloves) Medications
Group organising an annual marathon race and a local bank	1	Money for general newborn care equipment (oxygen concentrators and incubators)

* Not all facilities received all the items and some items pertain more to the provision of newborn services and are not directly required for KMC

** A not-for-profit organisation established in 1998 with Uganda and Nepal as initial target countries. Its mission is to make a positive difference to the lives of children in the developing world. (http://www.isisgroup.org/indexfound.html)

In all facilities there was staff that had been trained in KMC, either as a stand-alone training or as part of a broader training in essential newborn care. According to some informants, the introduction to KMC was brief but it resonated: “*Before, I had no idea KMC could save a baby*.” A total of 262 health workers were reportedly trained in KMC both on and off-site. However within the last 3 years, only 22% of the staff reportedly trained in KMC were still working with newborns at the time of the evaluation, due to attrition and staff rotation.

### KMC practice and documentation

Initiation of KMC practice for babies varied across the assessed health facilities. Decision to initiate KMC was made by the doctors (8 facilities) and jointly with nurses in 7 of these facilities. In the other 3 facilities nurses made the decision to initiate the baby on KMC. While verbal education was reported to be provided to mothers at and after initiation of KMC, information on preparation for KMC during antenatal care was not elicited. Some informants appeared to be unclear about the distinction between intermittent and continuous KMC. In only 3 facilities with a special KMC space was KMC practised for more than 20 hours per day. In 2 facilities records could be provided for babies receiving intermittent KMC and for how long per session. Mothers in 3 facilities were diligent in practising KMC and there was evidence of some KMC in 4 other facilities. In 1 facility there was little evidence of any KMC being practiced and in 3 instances mothers’ compliance with KMC could not be probed as there were no preterm babies at the time of the visit and no records to verify practice.

In 2 facilities mothers were not allowed to leave the KMC space as a measure of infection prevention while in 1 hospital mothers brought their babies to a heated nursery when going out. In 8 of the facilities mothers were allowed to have a guardian or companion while in the other sites access was strictly controlled.

Support for exclusive breastfeeding is important for the success of KMC practice. Only one of the hospitals visited had officially been designated as baby-friendly around 2005 and it had not been subsequently reassessed. Two hospitals had been assessed towards the end of 2011 but had not heard the outcome of the assessment. Three hospitals could produce a written feeding policy, whereas 6 hospitals had a feeding job aid for calculating the volumes of feeds that was displayed on the wall. There was written evidence of expressed breastmilk feeding in only 3 facilities.

Babies’ response to KMC and feeding was also monitored and in 7 facilities it was indicated that they weighed the babies regularly. Four facilities weighed once per day, 2 on alternate days and 1 weekly. Only one hospital did not have a scale while 3 of them had a mechanical one. Change in weight of babies was benchmarked on admission weight and discharge weight was also taken and recorded in varying types of documents including nursing and doctors’ notes, the baby's file (e.g. observation charts), the mother's chart, the KMC register and the discharge form. There was evidence of record keeping in all health facilities including locally-adapted KMC registers. In 4 hospitals evidence was found of doctors’ daily notes, which could include a prescription for the commencement of KMC. Two facilities recorded KMC on the discharge letter/form and one in the baby's health booklet.

### Facility discharge and follow-up care

Decision to discharge a baby from the health facility was a joint effort between doctors and nurses in 8 of the health facilities while in 3 of the facilities it was solely made by nurses. There were differences in the reported criteria for discharge followed and documentation was lacking. Only 2 facilities had discharge checklists or procedures.

Four facilities had evidence of a good follow-up system and could provide records of visits. Two could provide some evidence of follow up, whereas in the 2 health centres and 3 district hospitals no evidence could be provided. Babies were followed up in either the neonatal unit/KMC space (n = 4), or the maternity ward (n = 3), or the paediatric outpatient clinic (n = 4). Only one hospital had a special premature clinic on Fridays. Keeping records for follow up was found to be of “good” quality in one facility whereas it was “average” for 6 facilities and deemed “poor” in 4.

Follow up at the facility where the baby had been born or had received KMC was reported to be done until the baby reached a specific weight or a specific age. Weights mentioned were 2 kg (n = 1), 2.5 kg (n = 5) and 3 kg (n = 2). However, estimates by informants on the percentage of babies returning for follow up varied between “*few*” in one health centre, 30 to 50% in 3 other facilities, 80 to 90% in 2 hospitals and 95% or more in 2 of the private, not-for-profit hospitals. The main reason given for poor follow-up rates was distance from the facility.

## Discussion

This is the first evaluation in Uganda focusing on KMC implementation and scale-up progress. This standardised methodology provides a general picture of progress towards institutionalisation of KMC practice in health facilities and provides information on that can be used for further scale up.

The study has a number of limitations. The interviews were limited to health facility clinical and management staff and it did not assess the views of mothers practicing KMC and therefore demand for KMC at the community level and barriers to accessing care were not explored. Given the absence of mother-baby dyads in KMC during the assessment, this is an important area for further qualitative research. While it did not aim to provide results generalizable to other institutions within Uganda or beyond, lessons could be drawn from the strengths and challenges experienced with the institutionalisation of KMC in these sites.

KMC services in Uganda are mainly at the early implementation stage without widespread evidence of integration into routine practice. Managerial and leadership support was found to be a critical factor in fostering progress to institutionalisation. This general picture is not different from other African countries implementing KMC [19]. While private support was deemed to be essential in setting up services and reaching a level of implementation quickly, district and facility level support is essential in order to institutionalise services beyond the scope of any one project or donor. The private-not-for-profit hospitals performed better than the government facilities. Access to additional resources, a longstanding tradition of training health workers as well as management support may have contributed to faster progress. These sites could be used to provide outreach or exchange visits with stronger links between the government sector and private facilities.

This evaluation raises important questions about the investment in training health workers. Less than one quarter of health workers trained in KMC were still caring for newborn babies just three years after training, either due to rotation to other wards, transfer, or attrition. Given Uganda's existing challenges with adequate human resources for health [11], this drain is costly and derails progress of institutionalisation. Standard protocols for on-the-job orientation to KMC could ease the demand of health workers being taken off-site for retraining but this requires ownership of head nurses and administration. Production and dissemination of job aids and guidelines for KMC practices in all health facilities conducting deliveries will support standardisation of KMC practice, alongside routine assessment against existing newborn care standards [20]. Standardisation of practice across facilities according to these standards is necessary, with adaptation only where necessary and provided it will not compromise quality of care.

KMC is a comprehensive package involving the act of positioning the baby, companionship, feeding support, monitoring, facility discharge with follow up care, and record keeping. The services offered on the ground reflect varying degrees of practice. Feeding is a cornerstone for weight gain and temperature maintenance in KMC practice and yet only 2 facilities had been designated as baby-friendly and few had specific protocols to support exclusive breastfeeding. Additionally, follow-up services were a major gap, requiring improvements in record keeping and linking to step-down facilities where discharged mothers are bringing their babies for follow-up care. In order to increase demand for services, the practice of KMC should be promoted along the continuum of care starting in pregnancy (at antenatal clinic visits and through home visits from Village Health Team members), especially for women at risk of preterm birth.

While some of the KMC training reported was standalone, further scale up of KMC appears to rest with integration into two fast-moving and well-resourced national programmes: Helping Breathe Plus and Integrated Community Case Management for childhood illness. The risk of integration is that the scope of training in KMC is limited given the breadth of material both of these packages cover through in-service training. This is consistent study findings where informants indicated that KMC introduction was limited and brief [17, 18].

All of the facilities known to be practicing KMC were located in Kampala, the two Central regions, Southwest and the East-Central region. While this has been advantageous for teaching and learning opportunities, it represents a highly inequitable distribution of KMC services. Different partners have piloted KMC implementation in a piecemeal fashion in different sites in the country, depending on local champions at the facility or district level to take services forward. Given Uganda's position as a global champion for preterm birth care [21] and the prominent focus on KMC in the country's recently launched Reproductive, Maternal, Newborn, and Child Health sharpened plan [22], there is a need for a national roll-out strategy which prioritises districts with the highest burden of preterm births and deaths. One way to gain prominence for KMC on district and national agendas is to ensure at least one KMC indicator is captured and reported through the routine health management information system.

## Conclusion

KMC provides a cost-effective opportunity to improve care within an overstretched healthcare system and give vulnerable newborns a better chance of survival and health. Yet even in better-resourced facilities closer to the capital city, KMC services are not instituted with consistent levels of quality and are often dependent on private partner support. With attention globally and in country, Uganda is in a unique position to accelerate access to and quality of health services for small babies across the country.
